# Dual influenza and pneumococcal vaccination was associated with lower short-term risks of all-cause and acute respiratory hospitalizations among the elderly in Shenzhen, China: a retrospective cohort study

**DOI:** 10.1080/22221751.2020.1854624

**Published:** 2020-12-10

**Authors:** Yawen Jiang, Zhaojia Ye, Daqin Chen, Yuelong Shu

**Affiliations:** a School of Public Health (Shenzhen), Sun Yat-sen University, Shenzhen, People’s Republic of China; b Shenzhen Center for Disease Control and Prevention, Shenzhen, People’s Republic of China

**Keywords:** Influenza, pneumonia, vaccine, elderly, risk, hospitalization

## Abstract

The present study evaluated the real-world effectiveness of influenza and pneumococcal dual-vaccination among Chinese elderly, the evidence on which was absent. Outpatient and inpatient claims databases from Jan 1, 2015 to Apr 1, 2017 of persons at least 60 years old in Shenzhen, China were merged with electronic records of influenza vaccines and 23-valent pneumococcal polysaccharide vaccines (PPSV23) from Oct 1, 2016 - May 31, 2017. Individuals who were vaccinated with influenza between Nov 1 and Dec 31, 2016 and received PPSV23 30 days within the date of influenza vaccination were defined as the vaccinated group. A control group consisted of individuals that received neither of the vaccines was constructed by matching on year of birth, sex, and district. The two outcomes were all-cause and acute respiratory hospitalizations. Difference-in-difference (DiD) logistic regressions that were proceeded with an entropy balancing (EB) process were used to analyse the effectiveness of dual-vaccination. A total of 48,116 eligible individuals were identified in the vaccinated group, which were matched by 93,692 individuals in the control group. The EB-DiD analyses estimated that dual-vaccination was associated with lower short-term risks of all-cause (odds ratio: 0.59, CI: 0.55-0.63) and acute respiratory (odds ratio: 0.49, CI: 0.41-0.59) hospitalizations.

## Introduction

While both influenza and invasive pneumococcal disease (IPD) are major public health threats in China [1,2], they are also among the most preventable infectious diseases [3,4]. To confine the impact of influenza epidemics, the World Health Organization (WHO) recommends annual influenza vaccination among the elderly [5]. Similarly, WHO and the United States Centers for Disease Control and Prevention (USCDC) recommend inoculation with 23-valent pneumococcal polysaccharide vaccine (PPSV23) among the elderly to minimize the impact of IPD [6]. The Chinese guidelines also encourage the use of both influenza vaccines and PPSV23 among individuals with chronic illness [6].

Despite ample efforts of the public health and academic communities, the uptake of both types of vaccines have been suboptimal in China [6–8]. Among other factors, financial barriers have hampered broad access to influenza vaccines and PPSV23 [8,9]. To lift such barriers among the elderly, the municipal government of Shenzhen, a city with over 13 million residents in Southern China, deployed a publicly financed influenza and PPSV23 dual-vaccination programme in 2016, which was a leap from the lack of access to both. Starting from Oct 31, 2016, the programme provided free access to trivalent inactivated influenza vaccines and PPSV23 for residents aged 60 years or older with Shenzhen census registry (hukou) or Shenzhen social health insurance. Although it has been documented in numerous countries and regions that such dual-vaccination programmes reduced the incidence of influenza, pneumonia, all-cause mortality, cardiovascular mortality, and all-cause hospitalizations among the elderly compared with no vaccination [5,10], there is still a paucity of such evidence for Shenzhen specifically and for Mainland China in general. To investigate the effectiveness of the dual-vaccination programme in Shenzhen, the present retrospective cohort study using real-world data was conducted.

One of the increasingly popular approaches to assess real-world effectiveness of influenza vaccine is test-negative case–control design [11], in which medically attended patients presenting themselves with influenza-like illness are classified as cases or controls depending on the laboratory confirmation of influenza infection. However, such an approach has not been implemented for the evaluation of the composite effects of influenza and pneumonia vaccines to our knowledge. Alternatively, cohort studies have been conducted to evaluate both influenza vaccine effectiveness (VE) alone and the effects of dual vaccination [11–14]. For example, a study in the US examined the comparative effectiveness of high-dose versus standard-dose influenza vaccines using administrative claims databases, which were among the most exploited data sources for observational comparative effectiveness research [15].

The present study, adding to the body of evidence on influenza and pneumococcal VE, used Shenzhen social health insurance claims databases to compare the effects of dual-vaccination programme with no vaccination on reducing all-cause and acute respiratory hospitalizations. We hypothesized that receiving both the influenza vaccine and PPSV23 was associated with lower risks of all-cause and acute respiratory hospitalizations.

## Methods

### Data source and study design

The study primarily drew upon the outpatient and inpatient administrative claims databases from Jan 1, 2015 to Apr 1, 2017 of persons at least 60 years old covered by social health insurance. These databases were used to construct vaccinated and control groups for comparison. To ascertain vaccination status, vaccination registry of influenza and PPSV23 among the elderly from Jan 1, 2016 through May 31, 2017 were provided by Shenzhen Center for Disease Control and Prevention (SZCDC) and merged with the claims databases. Based on the epidemic of the 2016–2017 flu season in Southern China and the temporal pattern of vaccination densities (Supplementary Table S1) [16], individuals who received influenza vaccination between Nov 1 – Dec 31, 2016 were identified initially, the date of which was set as the index date. Additional inclusion criteria were that the individual had a valid pseudo-ID and received a PPSV23 vaccine 30 days before or after the index date.

**Figure 1.**
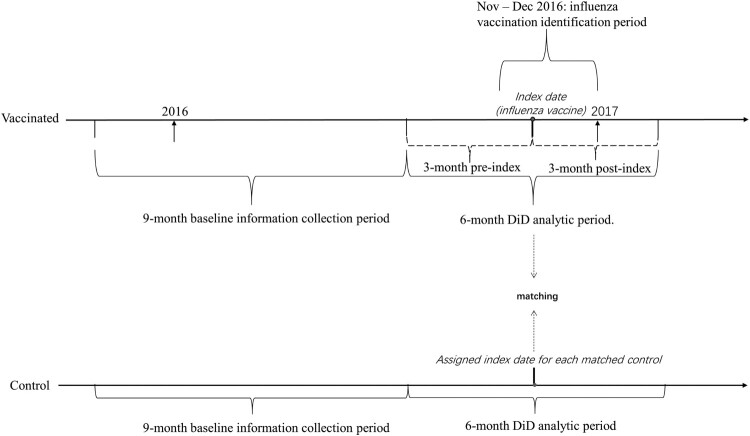
Time periods of each observation in the vaccinated and control groups. The matching of potential individuals for the control group and further selection of control group individuals is as following. (1) Vaccinated individuals were matched to all inpatient and outpatient medical occurrences of controls using age at index date/medical occurrence, sex, and district. (2) The index date of each vaccinated individual were assigned to the matched potential controls. (3) Four controls among the matched potential controls were randomly selected for each vaccinated individual. (4) Some individuals in the control group might have multiple appearances because they were matched to more than one vaccinated individual with distinct index dates. To resolve this, a distinct set of control observations were further filtered by keeping the observation of each control with the earliest index date.

Anchoring on the index date, a baseline period (the 12th month to the 3rd month before index), a 3-month pre-index period, and a 3-month post-index period were created. The baseline period was used to collect clinical characteristics of the individuals whereas the 3-month pre-index and post-index periods comprised a 6-month analytic period to conduct difference-in-difference (DiD) analyses which are elaborated in the statistical analysis section. Figure 1 schematizes the design of the study.

Following the identification of eligible vaccinated individuals, a control group was constructed by matching individuals in the claims database who received neither of the vaccines at any time in the vaccination registry. The matching and selection of controls were conducted in four steps. First, potential controls were matched to the vaccinated individuals on year of birth, sex, and district (sub-city jurisdictions). Second, the index date of each vaccinated individual was assigned to the matched non-vaccinated individuals. Third, four controls of each vaccinated individual were randomly chosen. Finally, the earliest occurrence of each non-vaccinated individual in the remaining control pool was selected if this individual was matched to more than one vaccinated person.

The present analysis used de-identified secondary data and was exempt from ethical reviews under local regulations.

### Intervention, outcomes, and covariates

The intervention in the present study was receiving both vaccines, defined as receiving a PPSV23 vaccination 30 days within a flu shot. The two outcomes were all-cause and acute respiratory hospitalizations. The latter was identified using ICD-10 diagnosis codes listed in Supplementary Table S2.

The covariates in the analyses were (1) demographic variables including age, sex, and district; (2) overall physical condition proxies including any baseline hospitalization, baseline inpatient costs, any baseline outpatient visits, and baseline outpatient costs; (3) baseline non-chronic respiratory illness including any flu or pneumonia hospitalizations, any acute upper respiratory infection hospitalizations, any lower respiratory infection hospitalizations other than flu and pneumonia, any hospitalizations due to other acute disorders of lung, any coughing-related hospitalizations, any hospitalizations due to abnormalities of breathing, any hospitalizations due to pain in throat and chest, any outpatient visits due to respiratory tract infection, and any outpatient visits due to coughing; and (4) baseline comorbidities including any hospitalizations due to lipoprotein metabolism disorders, any hospitalizations due to coronary heart disease, any hospitalizations due to hypertension, any hospitalizations due to chronic lower respiratory diseases, any hospitalizations due to fatty liver, any hospitalizations due to type 2 diabetes mellitus, any outpatient visits due to arthritis, any outpatient visits due to hyperlipidemia or atherosclerosis, any outpatient visits due to cerebrovascular diseases and/or sequelae, any outpatient visits due to hypertension, any outpatient visits due to chronic obstructive pulmonary diseases, and any outpatient visits due to diabetes. The hospitalization variables in covariate categories (3) and (4) were based on ICD-10 diagnosis codes (Supplementary Table S2) whereas the outpatient occurrences were based on combinations of keywords (e.g. “inflammation” and “throat”; Supplementary Table S3) in the diagnosis field because outpatient visits were not billed using ICD-10 codes in China.

### Statistical analysis

T-tests and $\chi^{2}$-tests were conducted for unadjusted comparisons of baseline characteristics and outcomes across groups. To estimate the effects of dual vaccination, the present study combined entropy balancing (EB) with DiD. EB resembles propensity score matching (PSM) in that it aims to balance covariates across two groups upfront instead of adjusting for covariates in multivariate regressions [17]. However, it also distinguishes itself from PSM with two key features. First, it directly balances each of the covariates by re-weighting each of the samples instead of matching on a single index [17]. Second, it allows the balance of both the first moment (e.g. mean) and the second moment (e.g. variance) [18]. For these reasons, it is potentially more efficient than PSM to control for observed confounding [19]. All abovementioned covariates were used in the EB process.

Despite its appealing properties, EB also shares the same defining pitfall of PSM – failing to account for unobserved confounding [20–22]. To supplement the EB approach in this regard, the EB process was sequenced by DiD analyses [23]. The DiD method, a well-established causal-inference approach in public health, specifically addresses two types of unobserved confounding, namely time-invariant cross-group confounding such as historical medical occurrence, and time-varying within-group confounding such as seasonal patterns [23–25]. A key assumption of DiD is that the two groups have parallel temporal trends of the outcomes of interest [23]. In other words, the topologies of the time series of the outcomes should be similar were there no interventions. To increase the validity of this assumption, the outcome variables in each month of the 3-month pre-index period were also included in the EB process, which allowed the pre-index temporal patterns of outcome incidence to not only parallel but also coincide. The DiD equation of the present analysis took the form:

(1) $$\begin{array}{c} Y_{\mathrm{it}}=\beta_{0}+\beta_{1}\times \mathrm{post}+\beta_{2}\times v+\beta_{3}\times \mathrm{post}\times v+\varepsilon_{\mathrm{it}}\; \end{array}$$

where $Y_{\mathrm{it}}$ was the outcome in period *t* for individual *i*, *t* ∈ *[pre, post]*, *v* was an indicator of whether individual *i* was in the vaccinated group, and *post* was an indicator of whether the current period of observation was a post-index period. Of note, *v* was a group indicator instead of a vaccination status indicator such that it did not indicate whether an individual was vaccinated in the current observation. As such, each individual had two observations in the regression. To illustrate, the first observation of an individual in the vaccinated group took the value 1 for *v* and 0 for *post*, and the second observation took the value 1 for both *v* and *post*. With the DiD specification, the coefficient of the interaction term, $\beta_{3}$, represented the intervention effect of interest, whereas $\beta_{1}$ and $\beta_{2}$ tested within-group temporal effect and underlying cross-group differential trends, respectively. Weighted logistic regressions using weights created from the EB process were carried out to estimate Equation (1) for both outcomes, following which the effects of vaccination were given by the odds ratios (ORs) of the interaction term. All analyses were conducted using SAS 9.4 and Stata 15, and *p*-values of <0.05 were considered statistically significant.

### Sensitivity analysis

If the estimates of VE from the DiD analyses were statistically significant, the E-value would then be calculated to quantify the minimum intensity of associations of an unobserved confounder with the intervention and an outcome, respectively, to be able to explain away the estimated effects [26].

### Role of the funding source

The funding source of the study had no role in study design, data collection, data analysis, data interpretation, or writing of the report. The lead author had full access to all the data in the study. The correspondents had final responsibility for the decision to submit for publication.

## Results

**Figure 2.**
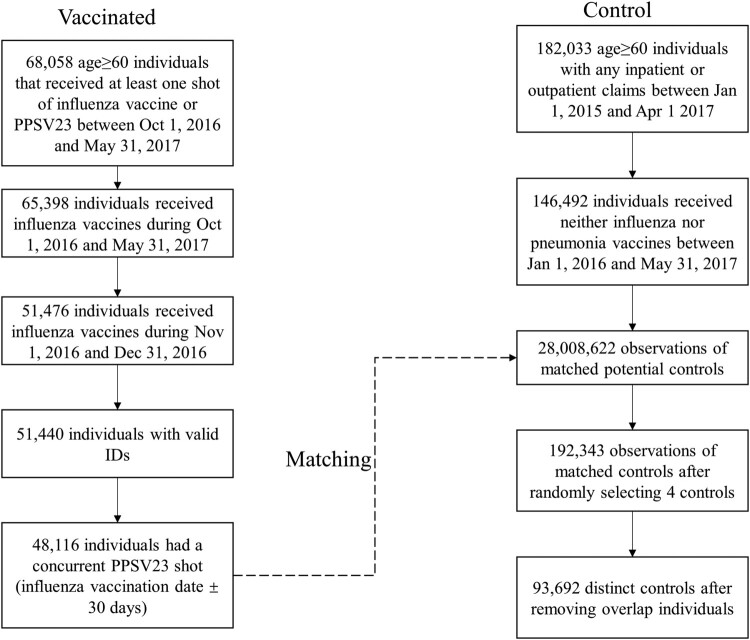
Flowcharts of sample selection in the vaccinated and control groups.

The selection process of vaccinated and control samples is presented in Figure 2. A total of 68,058 elderly individuals in Shenzhen received either or both of influenza and PPSV23 vaccines between Oct 1, 2016 – May 31, 2017, of which 65,398 received influenza vaccines. After restricting the samples to those received influenza vaccines between Nov 1 – Dec 31, 2016, documented with valid pseudo-IDs, and had concurrent PPSV23 vaccination within 30 days of the index date, 48,116 elderly individuals were included in the vaccinated group. In the meantime, there were 146,492 elderly individuals in the claims databases who received neither of the vaccines during Oct 1, 2016 – May 31, 2017. The first step of matching identified 28,008,622 observations as potential controls. After randomly selecting four matched controls for each vaccinated individual, 192,343 observations remained. Finally, 93,692 individuals were identified as controls after removing duplicate pseudo-IDs.

The vaccinated individuals were 69.6 years old [standard deviation (SD): 6.85)] on average and were 54.71% female. The baseline demographic and clinical characteristics of the vaccinated and control groups are described in Table 1. Uncertainty estimates were represented using variance instead of SD to be compliant with the output of EB. The individuals in the control group had systematically worse physical conditions in the baseline period. Specifically, the control group had significantly higher percentages of all types of medical occurrences in the covariate list during the baseline period except cough-related hospitalizations. For example, the percentages of individuals that had any baseline hospitalizations and outpatient visits in the control group were 21.93% and 51.62%, respectively, whereas the corresponding percentages in the vaccinated group were 13.40% and 39.30%. Also, the control group individuals had significantly higher mean baseline inpatient costs (CN¥ 5965, SD: 27487 vs. CN¥ 2187, SD: 10712; *p*<0.001) and outpatient costs (CN¥ 1420, SD: 4822 vs. CN¥ 865, SD: 3297; *p*<0.001). After re-weighting using EB, the two groups were balanced on each of the covariates and did not have significant difference in any of the characteristics.

**Table 1.** Demographic and baseline clinical characteristics of the vaccinated and control groups before and after entropy balancing.

	Control group before balancing (*N*=93,692)			Control group after balancing (weighted *N*=48,116)			Vaccinated group (*N*=48,116)	
	% or mean	variance	*p*-value of testing against the vaccinated group	% or mean	variance	*p*-value of testing against the vaccinated group	% or mean	variance
Age at index	70.0	55.0	<0.001	69.6	46.9	0.997	69.6	46.9
Female	51.52%	0.2498	<0.001	54.71%	0.2478	1.000	54.71%	0.2478
District			<0.001			1.000		
Guangming	2.62%	0.0255		4.22%	0.0404		4.22%	0.0404
Nanshan	12.86%	0.1121		13.09%	0.1138		13.09%	0.1138
Pingshan	1.54%	0.0152		1.42%	0.0140		1.42%	0.0140
Dapeng	1.22%	0.0121		1.63%	0.0161		1.63%	0.0161
Bao’an	10.77%	0.0961		10.33%	0.0926		10.33%	0.9262
Yantian	1.69%	0.0166		1.93%	0.0189		1.93%	0.0189
Futian	33.76%	0.2236		29.54%	0.2082		29.54%	0.2082
Luohu	20.95%	0.1656		19.96%	0.1597		19.96%	0.1597
Longhua	3.08%	0.0298		4.13%	0.0396		4.13%	0.0396
Longgang	11.50%	0.1018		13.75%	0.1186		13.75%	0.1186
Any baseline hospitalizations	21.93%	0.1712	<0.001	13.40%	0.1161	1.000	13.40%	0.1161
Any baseline outpatient visits	57.62%	0.2442	<0.001	39.30%	0.2386	1.000	39.31%	0.2386
Baseline inpatient costs	¥5,965	7.56E+08	<0.001	¥2,187	1.15E+08	1.000	¥2,187	1.15E+08
Baseline outpatient costs	¥1,420	2.33E+07	<0.001	¥865	1.09E+07	1.000	¥865	1.09E+07
Respiratory illness in the baseline period (not including chronic respiratory diseases)								
Any baseline flu or pneumonia hospitalizations	1.83%	0.0179	<0.001	0.93%	0.0092	1.000	0.93%	0.0092
Any baseline acute upper respiratory infection hospitalizations	0.63%	0.0062	<0.001	0.34%	0.0034	1.000	0.34%	0.0034
Any baseline acute lower respiratory infection hospitalizations other than flu and pneumonia	0.52%	0.0052	<0.001	0.35%	0.0034	1.000	0.35%	0.0034
Any baseline hospitalizations due to other disorders of lung	2.19%	0.0214	<0.001	0.80%	0.0079	1.000	0.80%	0.0079
Any baseline cough-related hospitalizations	0.14%	0.0014	0.192	0.11%	0.0011	1.000	0.11%	0.0011
Any baseline hospitalizations due to abnormalities of breathing	0.38%	0.0038	<0.001	0.19%	0.0019	1.000	0.19%	0.0019
Any baseline hospitalizations due to pain in throat and chest	0.24%	0.0024	0.001	0.13%	0.0013	1.000	0.13%	0.0013
Any baseline outpatient visits due to respiratory tract infection	19.21%	0.1552	<0.001	17.13%	0.1419	1.000	17.13%	0.1419
Any baseline outpatient visits due to coughing	3.46%	0.0334	<0.001	2.75%	0.0268	1.000	2.75%	0.0268
Comorbid conditions in the baseline period								
Any baseline hospitalizations due to lipoprotein metabolism disorders	4.56%	0.0435	<0.001	2.92%	0.0284	1.000	2.92%	0.0284
Any baseline hospitalizations due to coronary heart disease	4.71%	0.0449	<0.001	2.78%	0.0270	1.000	2.78%	0.0270
Any baseline hospitalizations due to hypertension	11.38%	0.1009	<0.001	6.69%	0.0624	1.000	6.69%	0.0624
Any baseline hospitalizations due to chronic lower respiratory diseases	2.77%	0.0269	<0.001	1.60%	0.0158	1.000	1.60%	0.0158
Any baseline hospitalizations due to fatty liver	2.39%	0.0233	<0.001	1.80%	0.0176	1.000	1.80%	0.0176
Any baseline hospitalizations due to type 2 diabetes	5.58%	0.0527	<0.001	3.18%	0.0308	1.000	3.18%	0.0308
Any baseline outpatient visits due to arthritis	4.10%	0.0394	<0.001	3.64%	0.0351	1.000	3.64%	0.0351
Any baseline outpatient visits due to hyperlipidemia or atherosclerosis	9.85%	0.0888	<0.001	6.99%	0.0650	1.000	6.99%	0.0650
Any baseline outpatient visits due to cerebrovascular diseases and sequelae	3.45%	0.0333	<0.001	2.41%	0.0235	1.000	2.41%	0.0235
Any baseline outpatient visits due to hypertension	22.49%	0.1743	<0.001	16.28%	0.1363	1.000	16.28%	0.1363
Any baseline outpatient visits due to COPD	0.65%	0.0064	<0.001	0.42%	0.0042	1.000	0.42%	0.0042
Any baseline outpatient visits due to diabetes	10.71%	0.0956	<0.001	7.55%	0.0698	1.000	7.55%	0.0698
Outcomes in the DiD analytic period								
Any hospitalizations in 3rd pre-index month	3.47%	0.0335	<0.001	1.82%	0.0179	1.000	1.82%	0.0179
Any hospitalizations in 2nd pre-index month	3.47%	0.0335	<0.001	1.94%	0.0190	1.000	1.94%	0.0190
Any hospitalizations in 1st pre-index month	3.86%	0.0371	<0.001	1.44%	0.0142	1.000	1.44%	0.0142
Any acute respiratory hospitalization in 3rd pre-index month	0.59%	0.0059	<0.001	0.28%	0.0028	1.000	0.28%	0.0028
Any acute respiratory hospitalization in 2nd pre-index month	0.59%	0.0059	<0.001	0.28%	0.0028	1.000	0.28%	0.0028
Any acute respiratory hospitalization in 1st pre-index month	0.59%	0.0058	<0.001	0.16%	0.0016	1.000	0.16%	0.0016

**Figure 3.**
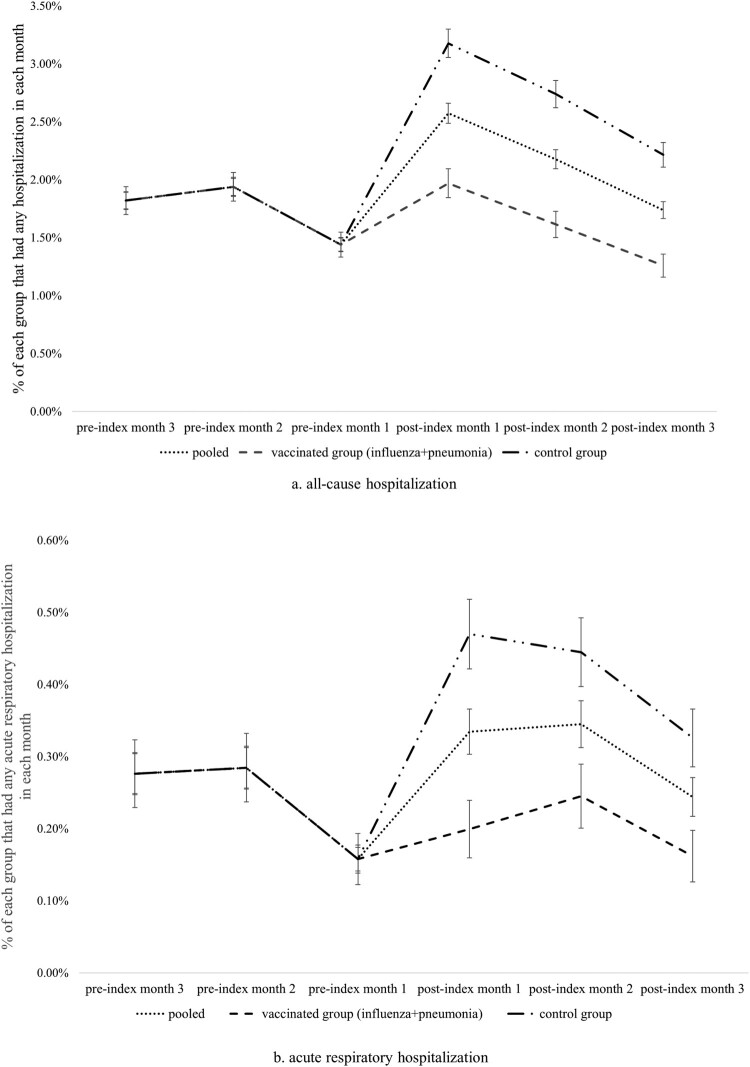
Percentages of samples that had all-cause and acute respiratory hospitalizations in each month of the difference-in-difference analytic time period.

The incidence rates of all-cause hospitalizations and acute respiratory hospitalizations by month in the pre-index and post-index periods within the DiD analytic time frame are depicted in Figure 3. As expected, the pre-index trends of both outcomes in the two groups were the same after EB. In both groups, the percentages of individuals that had any all-cause hospitalizations (Figure 3(a)) in the 3rd, 2^nd^, and 1^st^ pre-index months were 1.82%, 1.94%, and 1.44%, respectively. Similarly, the percentages for acute respiratory hospitalizations (Figure 3(b)) were 0.28%, 0.28%, and 0.16%. However, the percentages of all-cause hospitalizations (3.18%, 2.74%, and 2.22% vs. 1.97%, 1.61%, and 1.26%) and acute respiratory hospitalizations (0.47%, 0.44%, and 0.33% vs. 0.20%, 0.25%, and 0.16%) in the 1^st^, 2^nd^, and 3rd post-index months were substantially higher among the control group than the vaccinated group, although the trends in the two groups remained visually parallel.

The results of the weighted logistic regression using DiD specifications are listed in Table 2. According to these estimates, the ORs of all-cause and acute respiratory hospitalizations associated with dual vaccination were 0.59 (95% CI: 0.55-0.63) and 0.49 (95% CI: 0.41-0.59), respectively. Hence, the estimates of dual vaccination effectiveness against the two outcomes were 41% (95% CI: 37–45%) and 51% (41 - 59%), respectively. Also, the ORs associated with the vaccinated group indicator were statistically insignificant at 1.00 (95% CI: 0.95-1.05) and 1.01 (95% CI: 0.89-1.14) in the analyses of the two outcomes, suggesting no evidence of cross-group difference in the underlying trends of the two outcomes before vaccination following the implementation of EB.

**Table 2.** Results of weighted logistic regressions using difference-in-difference specifications.

	All-cause hospitalization	Acute respiratory hospitalization
Post × vaccinated	0.59*** (0.55–0.63)	0.49*** (0.41–0.59)
Post	1.56*** (1.51–1.62)	1.70*** (1.55–1.86)
Vaccinated	1.00 (0.95–1.05)	1.01 (0.89–1.14)
E-value to explain away post × vaccinated [point estimate (upper bound)]^a^	2.80 (2.55)	3.51 (2.80)

Results are presented as odds ratios (95% confidence intervals) unless otherwise specified.

^a^ The value for upper bound is the E-value required to generate an upper bound of a confidence interval that would be greater than 1.00.

*p<0.05.

**p<0.01.

***p<0.001.

The E-values to explain away the ORs in the two DiD analyses were 2.80 and 3.51, respectively, whereas the corresponding E-values of the upper bounds of 95% CIs were 2.55 and 2.80.

## Discussion

In the present study, we used claims databases to conduct a retrospective cohort study on the effectiveness of an influenza and PPSV23 dual-vaccination programme among the elderly in Shenzhen, China during the 2016–2017 flu season. The results of the study suggested that receiving both the trivalent influenza vaccine and PPSV23 was associated with a 41% lower risk of all-cause hospitalization and a 51% lower risk of acute respiratory hospitalization in the short term. To our knowledge, this is the first study that documented the real-world effectiveness of dual influenza and PPSV23 vaccination among elderly population in Mainland China. Previous cohort studies in other countries and regions have documented the effectiveness of dual influenza and pneumococcal vaccination to reduce the risk of all-cause, respiratory, and non-respiratory hospitalizations [27,28]. Our findings from the present cohort study concurs with the existing evidence in literature.

These findings entail important policy implications. Both influenza and IPD pose as major public health threats to the Chinese population, causing not only excess mortality but also immense medical costs [1,29]. Whereas a sizeable proportion of the disease burden related to influenza and IPD may be preventable using vaccines, the vaccination rates in China have not been encouraging [7]. The evidence from the present study indicate that implementing influenza and PPSV23 dual-vaccination programmes may be a viable approach to reduce population disease burden of the two conditions. With the potential co-circulation of COVID-19 and influenza [30], the prevention of influenza and IPD among the elderly could be more important than ever from both the individual and the societal perspectives given the disproportionate amount of hospitalizations and death related to COVID-19 among the elderly [30].

Although the present study established evidence on the effectiveness of dual vaccination, it may be rightfully argued that the benefit of such programmes should be weighed against their costs. Hence, future studies should shed light on the cost-effectiveness of influenza and pneumococcal vaccines among Chinese elderly. Information on the economic profiles of the dual-vaccination programme will further support the decision making related to immunization programmes and may insight on the reasonable range of prices that the government should pay for the vaccines.

The E-values of the ORs and their upper bounds suggest that the results were relatively robust to confounding bias. Specifically, a confounder needed to associate with being dual-vaccinated with a minimum risk ratio of 2.55 and associate with all-cause hospitalization with the same magnitude to sufficiently counteract the estimated VE on all-cause hospitalization. Similarly, the minimum risk ratio for a confounder to associate with both being dual-vaccinated and acute respiratory hospitalization to dissolve the corresponding VE was 2.80.

The present study has several strengths. First, our study included over 140,000 individuals in the cohort, making it one of the largest to evaluate VE in China. Second, our study engaged both EB and DiD methods to compare dual vaccination with no vaccination, accounting for not only observable but also several types of unobserved confounding. Conventionally, PSM has been extensively used in the evaluation of VE in cohort studies [31,32]. EB is a relatively new method that improves on PSM by reserving the covariate-balancing properties of PSM while enhancing the precision of balancing and reducing the uncertainty of estimates [17,18,22]. Following the EB process, the pre-index gradient in health conditions across the two groups was flattened. For example, the control group had higher pre-index rates of all-cause and flu or pneumonia hospitalizations, indicating relatively unhealthy states among the control group. However, the EB process managed to bring abreast the two groups on these fronts by equalizing all pre-index health proxies such that the two groups became more comparable intuitively. On top of that, DiD further reduced bias by cancelling out confounders within individuals across time periods [24]. Finally, the vaccination history information was ascertained using the electronic registry of SZCDC, minimizing the chances of exposure misclassification.

Several limitations must be noted when interpreting the results. As with most observational studies, the present study might be subject to residual confounding even with the joint implementation of EB and DiD. For example, potential confounders that were time-varying during the 6-month DiD analytic period such as the use of prescription medications could not be captured. Second, the present study only included data for the 2016–2017 flu season, thereby undermining its generalizability. Third, that only trivalent influenza vaccines were vaccinated through the programme could further weaken the generalizability of the study. Fourth, potential waning effects of the vaccines could not be investigated since only three months of claims data in 2017 were available. Fifth, the two outcomes we used might not have accurately reflected the true VE. All-cause hospitalization lacked specificity whereas acute respiratory hospitalization might underrepresent the events that could have been prevented by influenza and pneumococcal vaccines. Finally, over 90% of those who received the influenza vaccine also received PPSV23 under the dual-vaccination programme, yet the number of individuals receiving either of the vaccines was minimal outside of the immunization programme. Therefore, it was infeasible to reliably compare dual-vaccination with influenza vaccination alone.

## Conclusion

Dual influenza and PPSV23 vaccination may reduce all-cause and acute respiratory hospitalizations among Chinese elderly. Expanding access to influenza and pneumococcal vaccines for 60 years or older individuals through immunization programmes should be considered in China.

## Supplementary Material

supplementary_materials.docx

## Data Availability

The data that support the findings of this study are available from the Shenzhen Medical Informatics Center. Restrictions apply to the availability of these data, which were used under a license for studies. Data are available from the authors upon reasonable requests for requestors who have permission from Shenzhen Medical Informatics Center.
