# Supplementary material for: Dual influenza and pneumococcal vaccination was associated with lower short-term risks of all-cause and acute respiratory hospitalizations among the elderly in Shenzhen, China: a retrospective cohort study
Source: Emerg Microbes Infect. 2020 Dec 10;9(1):2578–87. doi: 10.1080/22221751.2020.1854624 (PMC7734018; doi:10.1080/22221751.2020.1854624)
Supplement: supplementary_materials.docx [file TEMI_A_1854624_SM7784.docx]

Table S1. Vaccination frequencies of influenza and PPSV23 by month between Oct 1, 2016 – May 31, 2017

|  | Influenza | | PPSV23 | |
| --- | --- | --- | --- | --- |
| month | frequency | percentage | frequency | percentage |
| 2016-10 | 406 | 0.62 | 389 | 0.61 |
| 2016-11 | 38763 | 59.15 | 35866 | 56.23 |
| 2016-12 | 12766 | 19.48 | 12872 | 20.18 |
| 2017-01 | 3401 | 5.19 | 3392 | 5.32 |
| 2017-02 | 2534 | 3.87 | 2413 | 3.78 |
| 2017-03 | 6897 | 10.52 | 6330 | 9.92 |
| 2017-04 | 763 | 1.16 | 1727 | 2.71 |
| 2017-05 | 1 | 0.00 | 792 | 1.24 |

Table S2. ICD-10 codes used in the identification of covariates and outcomes in the inpatient setting

| Item NO. | condition | code |
| --- | --- | --- |
| 1 | flu or pneumonia hospitalizations | J09-J18 |
| 2 | acute upper respiratory infection hospitalizations | J00-J06 |
| 3 | lower respiratory infection hospitalizations other than flu and pneumonia | J20-J22 |
| 4 | hospitalizations due to other acute disorders of lung | J98.4 |
| 5 | coughing-related hospitalizations | R05 |
| 6 | hospitalizations due to abnormalities of breathing | R06 |
| 7 | hospitalizations due to pain in throat and chest | R07 |
| 8 | Acute respiratory hospitalizations | Any of items 1-7 |
| 9 | hospitalizations due to lipoprotein metabolism disorders | E78 |
| 10 | hospitalizations due to coronary heart disease | I25 |
| 11 | hospitalizations due to hypertension | I10 |
| 12 | hospitalizations due to chronic lower respiratory diseases | J40-J47 |
| 13 | hospitalizations due to fatty liver | K76.0 |
| 14 | hospitalizations due to type 2 diabetes mellitus | E11 |

Table S3. Combinations of key words used in the identification of covariates in the outpatient setting

| Item NO. | condition | Key words in the diagnosis data field | Key words in simplified Chinese characters |
| --- | --- | --- | --- |
| 1 | outpatient visits due to respiratory tract infection | (“infection” or “inflammation) and (“respiratory” or “bronch”/“trach” or “pulmonary”/“lung” or “pharyn” or “laryn” or “nasal”/“nose” | (“染” or “炎”) and (“呼” or “气” or “肺” or “咽” or “喉” or “鼻”) |
| 2 | outpatient visits due to coughing | “cough” | “咳” |
| 3 | outpatient visits due to arthritis | “arthritis” | “关节炎” |
| 4 | outpatient visits due to hyperlipidemia or atherosclerosis | (“high”/“hyper” and “lipid” and “blood”) or (“high”/“hyper” and “cholesterol”) or “atherosclerosis” | (“高” and “脂” and “血”) or (“高” and “胆固醇”) or “粥样硬化” |
| 5 | outpatient visits due to cerebrovascular diseases and/or sequelae | “cerebro” + “vascular” | “脑”+“血” |
| 6 | outpatient visits due to hypertension | “hypertension” | “高血压” |
| 7 | outpatient visits due to chronic obstructive pulmonary diseases | “pulmonary”/“lung” + “obstruct” | “肺”+“阻” |
| 8 | outpatient visits due to diabetes | “diabetes” | “糖尿” |
